# A Case Report of Extensor Pollicis Longus Tendon Rupture: Delayed Diagnosis in a Professional Rugby Player

**DOI:** 10.7759/cureus.71265

**Published:** 2024-10-11

**Authors:** Siddhant P Nayak, Molly Papadopoullos, Niyam Amanullah, Christos Kitsis, Cecilia Brassett, Neil Ashwood

**Affiliations:** 1 Physiology, Development and Neuroscience, Human Anatomy Centre, University of Cambridge, Cambridge, GBR; 2 Trauma and Orthopaedics, University Hospitals of Derby and Burton, Burton, GBR; 3 Trauma and Orthopaedics, University Hospitals of Derby and Burton NHS Foundation Trust, Derby, GBR

**Keywords:** distal end radius fracture, extensor indicis tendon transfer, pulvertaft weave technique, screw abrasion with tendon, upper limb orthopedic surgery, volar plating

## Abstract

Spontaneous rupture of the extensor pollicis longus (EPL) tendon has been reported to be uncommon. Several possible causes and precipitating factors have been reported for this rupture, including prior surgery to the distal radius. We report the case of an 18-year-old male professional athlete who presented with an inability to extend their left thumb. They presented with this condition after open reduction and internal fixation (ORIF) for a fractured left distal radius. Findings were consistent with the rupture of the EPL tendon. The typical presentation of such a rupture is one to four months after ORIF surgery, a shorter delay than seen in the present case, which exceeds one year. They underwent surgical tendon transfer using the extensor indicis tendon, and a dorsally protruding surgical screw was removed from the volar aspect of the radius. In this case, we suggest that repetitive friction from the protruding surgical screw, following volar plating of a distal radius fracture, may have caused abrasion of the EPL tendon. This may have predisposed it to rupture. This can occur long after the placement of a volar plate and must be considered as a potential cause of an EPL tendon rupture.

## Introduction

Volar plating is a common orthopedic technique used to treat fractures of the distal radius (one of two bones comprising the forearm) [1]. This procedure involves securing a metal plate to the front (volar side) of the wrist using screws, which helps stabilize the fracture [2]. However, in some cases, screws may penetrate the back (dorsal) side of the wrist, leading to complications like tendon rupture. This risk has been reported to be up to 12.5% [3]. The rupture is believed to be due to mechanical abrasion between the penetrating screws and the extensor tendons. An extensor tendon particularly at risk is the extensor pollicis longus (EPL) tendon [1,4].

EPL is a deep muscle at the back of the forearm and hand. It originates from the posterior shaft of the distal ulna and the interosseous membrane. It forms a tendon prior to crossing the wrist joint, changing the angle at Lister’s tubercle to insert at the distal phalanx (last digit) of the thumb [5]. The tendon’s position in the third extensor compartment makes it vulnerable to impingement from screws in volar plating [1]. This places the tendon at risk of rupture, with rupture leading to an inability to extend the thumb. This can impair activities of daily living, such as writing. In this report, we describe a case of EPL tendon rupture 15 months after volar plating for a distal radius fracture.

This case was previously presented as a poster at the 2024 British Association of Clinical Anatomists Summer Meeting on 2nd July 2024. Written informed consent for imaging and publication was obtained from all participants in this case report.

## Case presentation

The patient was an 18-year-old professional male rugby player who presented to the clinic in May 2024 with an inability to extend the left thumb, resulting in an abnormal thumb posture and poor grip. He had previously sustained a fractured left distal radius for which an open reduction and internal fixation (ORIF) was performed in March 2023, more than a year prior to presentation. There were no other predisposing factors.

**Figure 1:**
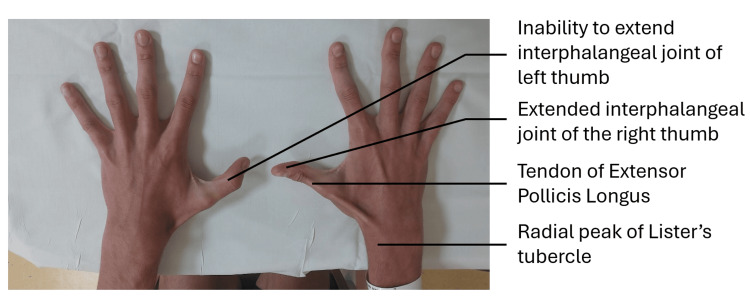
The patient was asked to extend both thumbs, lifting them off a flat surface. His inability to do so on the left due to a ruptured EPL tendon is clearly shown. EPL, extensor pollicis longus

**Figure 2:**
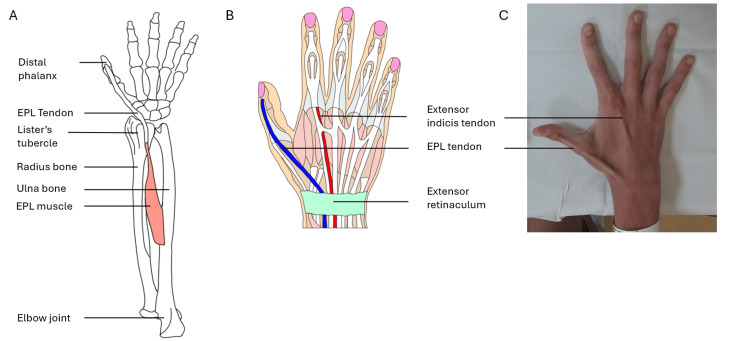
Diagrams and images to show positions of EPL and extensor indicis proprius on the posterior (back of the) forearm and hand. A) Diagram to show the position of EPL on the skeleton of the forearm and hand. B) Diagram to show the position of EPL and extensor indicis proprius relative to other soft tissues of the hand. C) Image to show surface anatomy of EPL and extensor Indicis Proprius. EPL, extensor pollicis longus

On clinical examination, the patient was unable to lift their thumb off a flat surface on the affected left side (Figure 1). This is called the retropulsion test, and this inability is reflective of an EPL tendon rupture. An intact EPL is responsible for the action of retropulsion due to its dorsal position (Figure 2).

**Figure 3:**
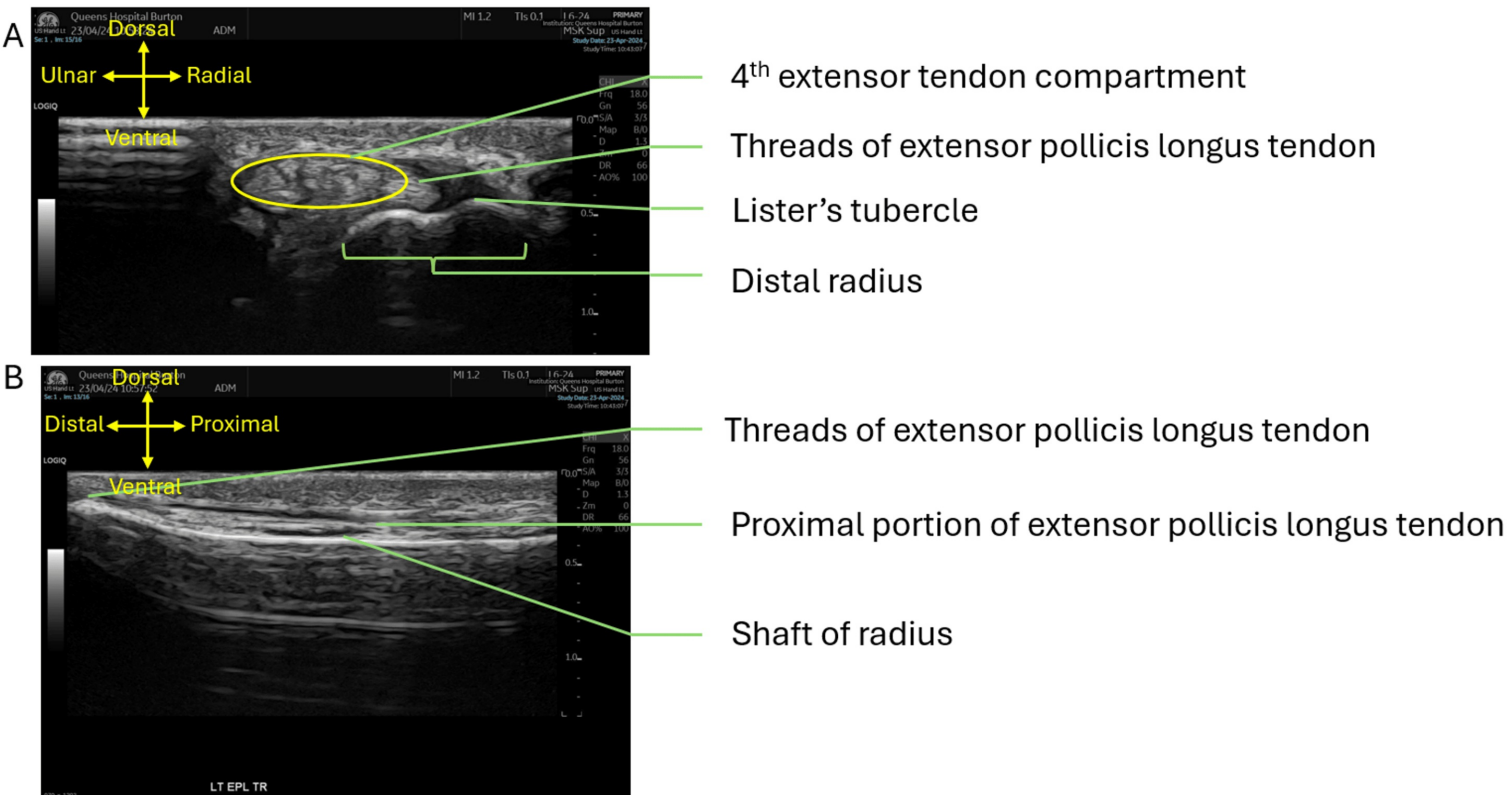
Ultrasound scan of distal left radius. A) Transverse view of the distal left radius at the level of Lister's tubercle. B) Longitudinal view of the distal left radius in line with the EPL EPL, extensor pollicis longus

**Figure 4:**
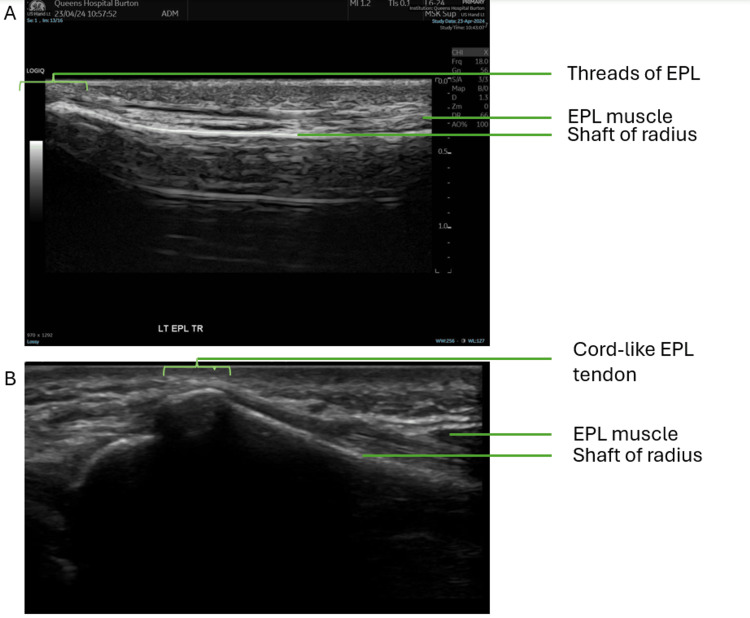
Longitudinal view of the distal left radius in line with the EPL tendon. A) Patient with a ruptured EPL tendon. B) Healthy volunteer with intact EPL tendon. EPL, extensor pollicis longus

Ultrasound scanning of the wrist and thumb using a linear array probe confirmed the diagnosis of a full-thickness tear of the EPL tendon at the level of the distal radius. During the scan, the EPL was less visible at the level of Lister’s tubercle, with only threads of EPL tendon fibers seen crossing over the second extensor compartment (see Figure 3 for scans of the patient and see Figure 4 for comparison with an intact EPL tendon). Beyond this rupture, distally in the thumb, the EPL appears intact and inserted normally at the base of the distal phalanx.

**Figure 5:**
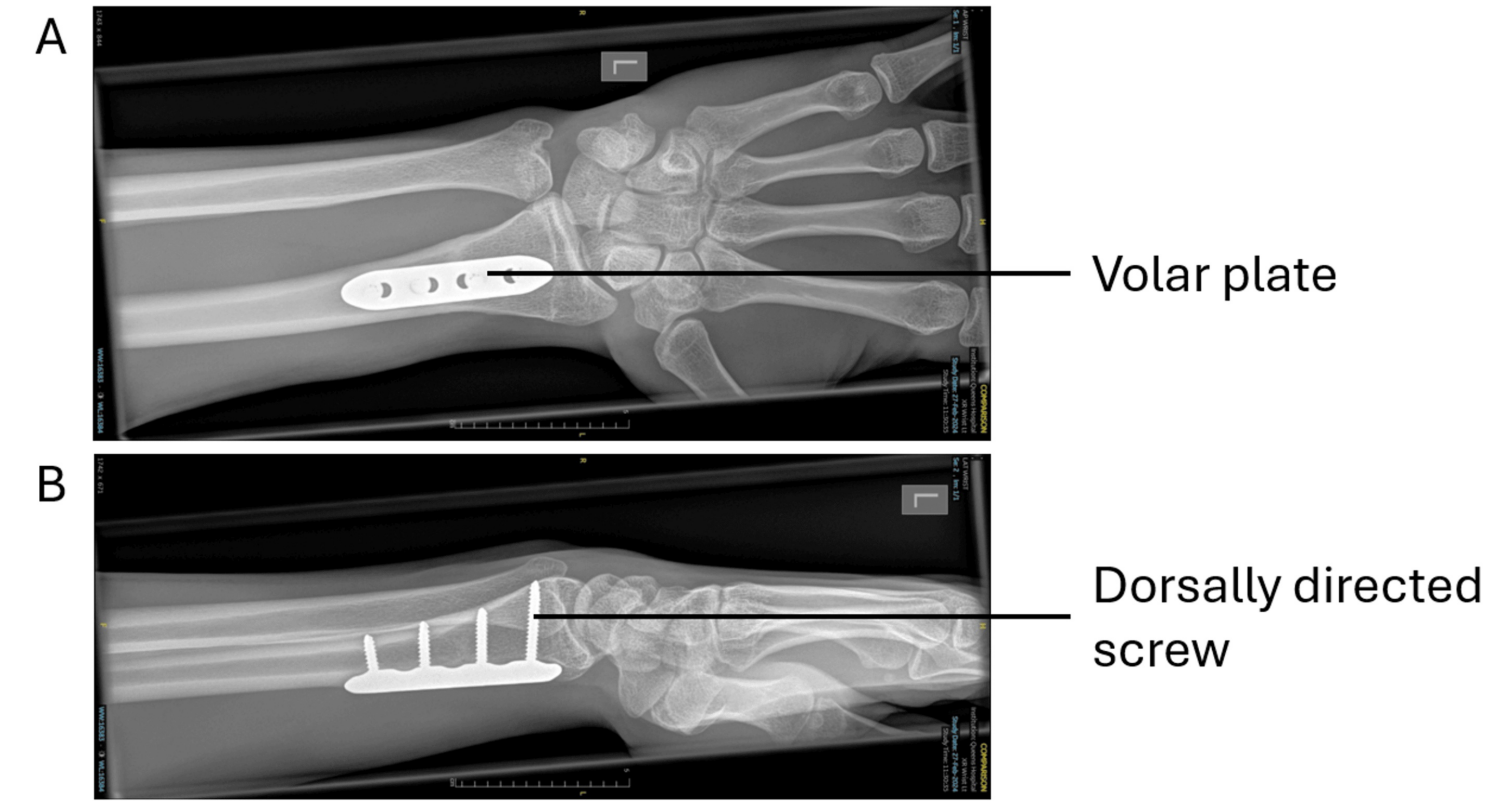
A) AP X-ray of the left wrist with a volar plate. B) Lateral X-ray of the left wrist with a volar plate.

**Figure 6:**
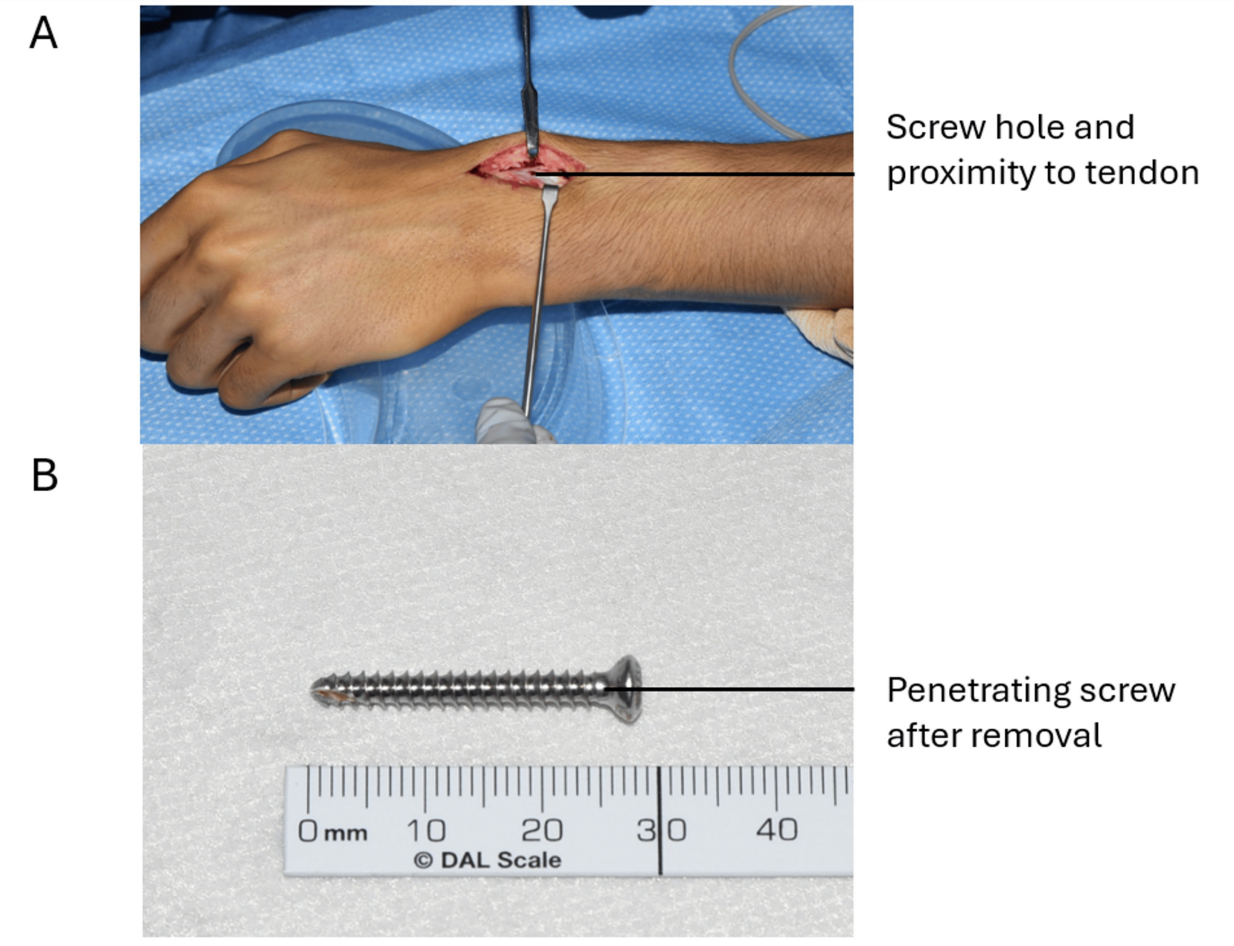
A) Incision to show the hole formed by a penetrating screw from the volar plate. B) 28 mm long penetrating screw after removal.

**Figure 7:**
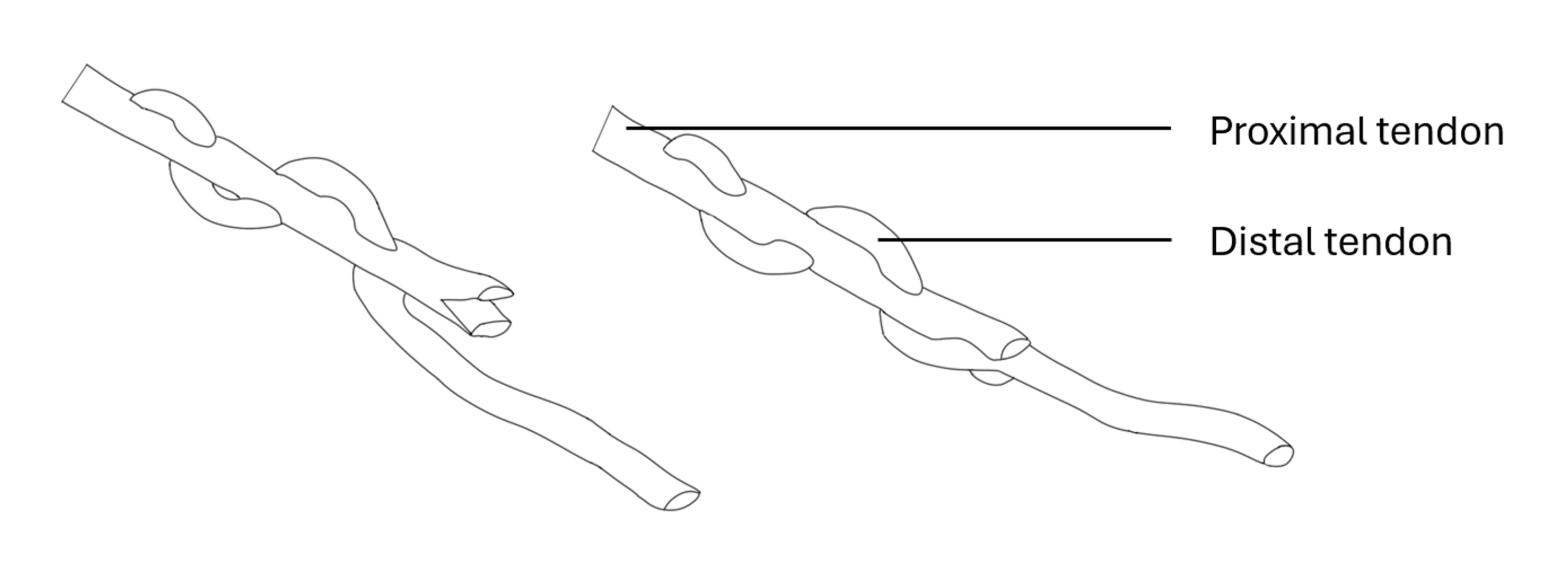
Pulvertaft weave with the distal tendon passing through the proximal tendon.

In May 2024, the patient underwent extensor indicis proprius (EIP) to EPL transfer. During surgery, the EPL was found to be absent at Lister’s tubercle. A 28 mm screw, which was prominent dorsally (see Figure 5), was removed through a volar incision (Figure 6). The EIP was mobilized from the left index finger through a separate incision and used to reconstruct the thinned and ruptured EPL with a Pulvertaft weave technique (Figure 7).

We suggest that repetitive friction from the protruding surgical screw may have caused abrasion of the tendon and predisposed it to rupture. We further suggest that mechanical abrasion must be considered as a potential cause of rupture in patients presenting with abnormal thumb movements after volar plating of distal radius fractures.

## Discussion

Spontaneous rupture of the EPL tendon is uncommon. Reports detail several possible causes and precipitating factors, which can be divided into mechanical and vascular causes [1]. Mechanical factors include fracture of the distal radius at Lister’s tubercle, bony spurs, subluxation of the distal ulna, and misplaced screws following surgical repair of a fractured distal radius [6]. Vascular factors include a decrease in the blood supply to the tendon, which could be due to inflammation such as tenosynovitis or systemic factors such as steroid treatment and rheumatoid arthritis.

Extensor tendon ruptures due to plating in distal radius fractures are more commonly seen with dorsal positioning rather than volar plating [7]. However, there are reports of volar plating leading to rupture, specifically in misplaced screws leading to penetration [8]. This risk has been reported to be up to 12.5% [3].

The present case of an EPL tendon rupture supports that this can occur following volar plating. The diagnosis was made after clinical testing by asking the patient to lift their thumb after placing palms on a flat surface (referred to as the retropulsion test). This diagnosis was confirmed by observing the course of the EPL tendon through musculoskeletal ultrasound, which is routinely performed when an EPL tendon rupture is suspected [9]. A rupture is confirmed by reduced sonographic visibility of the tendon from its typical location.

Diagnosis can be delayed as the patient may only experience subtle weakness and tenderness prior to rupture. Furthermore, the identification of the etiology was delayed as mechanical abrasion by a penetrating screw is a less well-known cause of an EPL tendon rupture. Rupture of the EPL tendon can occur long after volar plating of the distal radius. Previously reported cases of rupture have occurred four weeks to four months after volar plating, both significantly sooner than the delay in the present case (15 months) [4].

While in this case a transfer using the extensor indicis proprius was performed to repair the ruptured tendon, other tendons can also be used, such as the tendon of extensor digiti minimi or palmaris longus. The use of extensor indicis proprius offers advantages as there is no significant functional loss upon removal and it is present in 96.5% of individuals [10]. However, it can be prone to variation in interconnections with other tendons, notably the EPL tendon [11]. The palmaris longus tendon can also be used, although this is absent in up to 22.4% of Caucasian individuals, with this figure varying between populations [12]. The extensor indicis proprius is, therefore, the tendon of choice for such a transfer and was used in this case.

## Conclusions

Rupture of the EPL tendon is a possible adverse outcome following volar plating for a comminuted fracture of the distal radius and can occur long after volar plating has been performed. This case highlights that mechanical abrasion of the tendon by a penetrating screw must be considered as a potential cause if a patient presents with EPL tendon rupture.

This adverse outcome can be treated surgically with a tendon transfer to restore the ruptured tendon. Tendons that can be used are the extensor digiti minimi, palmaris longus, and extensor indicis proprius tendon. Each offers its own advantages and disadvantages, but as the extensor indicis proprius tendon is present more reliably and is more suitably positioned, it was used in this case.
